# Endoplasmic reticulum stress in innate immune cells - a significant contribution to non-alcoholic fatty liver disease

**DOI:** 10.3389/fimmu.2022.951406

**Published:** 2022-07-22

**Authors:** Liangliang Zhou, Haiyuan Shen, Xiaofeng Li, Hua Wang

**Affiliations:** ^1^ Department of Oncology, The First Affiliated Hospital of Anhui Medical University, Hefei, China; ^2^ Inflammation and Immune Mediated Diseases Laboratory of Anhui Province, Anhui Medical University, Hefei, China; ^3^ Anhui Institute of Innovative Drugs, School of Pharmacy, Anhui Medical University, Hefei, China

**Keywords:** non-alcoholic fatty liver disease, immune cells, unfolded protein response, endoplasmic reticulum stress, hepatic steatosis

## Abstract

Liver disease and its complications affect millions of people worldwide. NAFLD (non-alcoholic fatty liver disease) is the liver disease associated with metabolic dysfunction and consists of four stages: steatosis with or without mild inflammation (NAFLD), non-alcoholic steatohepatitis (NASH), fibrosis, and cirrhosis. With increased necroinflammation and progression of liver fibrosis, NAFLD may progress to cirrhosis or even hepatocellular carcinoma. Although the underlying mechanisms have not been clearly elucidated in detail, what is clear is that complex immune responses are involved in the pathogenesis of NASH, activation of the innate immune system is critically involved in triggering and amplifying hepatic inflammation and fibrosis in NAFLD/NASH. Additionally, disruption of endoplasmic reticulum (ER) homeostasis in cells, also known as ER stress, triggers the unfolded protein response (UPR) which has been shown to be involved to inflammation and apoptosis. To further develop the prevention and treatment of NAFLD/NASH, it is imperative to clarify the relationship between NAFLD/NASH and innate immune cells and ER stress. As such, this review focuses on innate immune cells and their ER stress in the occurrence of NAFLD and the progression of cirrhosis.

## Introduction

Liver disease is a major medical problem for human health. Non-alcoholic fatty liver disease (NAFLD) describes a range of liver conditions characterized by metabolic abnormalities, a global epidemic that seriously endangers people’s health and has become the most prevalent liver disease worldwide (1). It is defined as steatosis in more than 5% of hepatocytes and associated with metabolic risk factors (especially obesity and type 2 diabetes), but is not associated with excessive alcohol consumption (≥30 grams per day for men and ≥20 grams per day for women) or other chronic liver diseases (2). In the US, NAFLD affects 3% to 6% of the population, and it is most prevalent in patients with metabolic diseases and obesity. Despite its importance, NASH is underestimated in clinical practice. It is estimated that 20% of patients with NASH will develop hepatic fibrosis, and fibrosis is the most important prognostic factor for the long-term outcomes of NASH and are associated with increased liver-specific and overall mortality (3). The number of cirrhosis cases worldwide increased by 74.5% from 1990 to 2017, with NAFLD accounting for 59.5% of the cases (4). According to the National Institutes of Health, NASH is anticipated to be the leading cause of liver transplantation in the US, with a mortality rate that is substantially higher than the general population or in patients without this inflammatory subtype of NAFLD (5). Since there is no effective treatment for cirrhosis, it is critical to manage the disease in its early stages. Despite the urgency of treatment for this range of diseases, the underlying causes of the disease remain unclear. Current studies suggest that multiple factors, including protein abnormalities in signal transduction pathways, insulin resistance, oxidative stress, inflammation, intestinal bacterial translocation, and environmental factors, could contribute to disease progression in NAFLD. Among these, we cannot ignore the factor of inflammation in particular.

The recently suggested nomenclature changes to metabolic-associated fatty liver disease (MAFLD) draw attention to the root cause of the disease. As the current subclassification of this widespread hepatic metabolic disease remains to be defined by an international consensus group, this review will consider the literature on pathogenesis and progression under the old nomenclature NAFLD. Obesity and adipose tissue insulin resistance cause ectopic fat accumulation in the liver, thereby impairing hepatic insulin signaling, provoking ER stress, mitochondrial dysfunction, and oxidative stress, and inducing inflammation. Liver damage from cirrhosis is usually irreversible, the good news is if cirrhosis is diagnosed and treated early, further damage may be prevented and, in exceptional circumstances, reversed. In NAFLD improvement or worsening of disease activity may be associated with the regression or progression of fibrosis, respectively. According to Paul Angulo’s clinical study and some meta-analyses, the survival rate of clinical patients with NAFLD is related to the severity of inflammation and fibrosis (6).

Although the pathogenesis of NAFLD is complex and incompletely understood, interestingly, recent evidence has implicated the ER in the development of steatosis, inflammation and fibrosis. It is widely recognized that ER is a multifunctional organelle in eukaryotes that is essential for protein maturation. The accumulation of lipids in hepatocytes increases the demand for protein processing by the ER, causing misfolded proteins to accumulate in the ER lumen (7). Excess misfolded or unfolded proteins provoke ER stress, and the unfolded protein response (UPR) is triggered to restore homeostasis (8). UPR, which is associated with membrane biosynthesis, insulin action, inflammation, and apoptosis, serves to restore ER homeostasis by reducing protein synthesis and increasing protein folding and clearance (8). ER stress is prominently displayed in inflammatory responses, including direct defense against microbial pathogens, production of pro-inflammatory cytokines, immunogenic cell death, metabolic homeostasis and maintenance of immune tolerance (9). During these processes, immune cells infiltrate the liver and release pro-inflammatory cytokines and immunomodulatory mediators that may worsen hepatocyte dysfunction, resulting in hepatocyte necrosis, hepatic steatosis, and fibrosis, which may result in NAFLD and NASH (10, 11). On the other hand, the conditions most conducive to ER stress-mediated disease progression may include chronic injury that induces persistent ER stress, which is associated with a reduced or impaired ability of the general immune response to mitigate inflammatory damage (12). At the onset of NASH, damaged hepatocytes release a variety of signals, including damage-associated molecular patterns (DAMPs) and pathogen-associated molecular patterns (PAMPs), which activate local and mobilized immune cells and trigger an immune response.

Therefore, the mechanisms that disrupt ER homeostasis in NAFLD and the role of ER stress on innate immune cells in the occurrence and development of NAFLD are gradually being explored in more detail.

## The unfolded protein response

**Figure 1:**
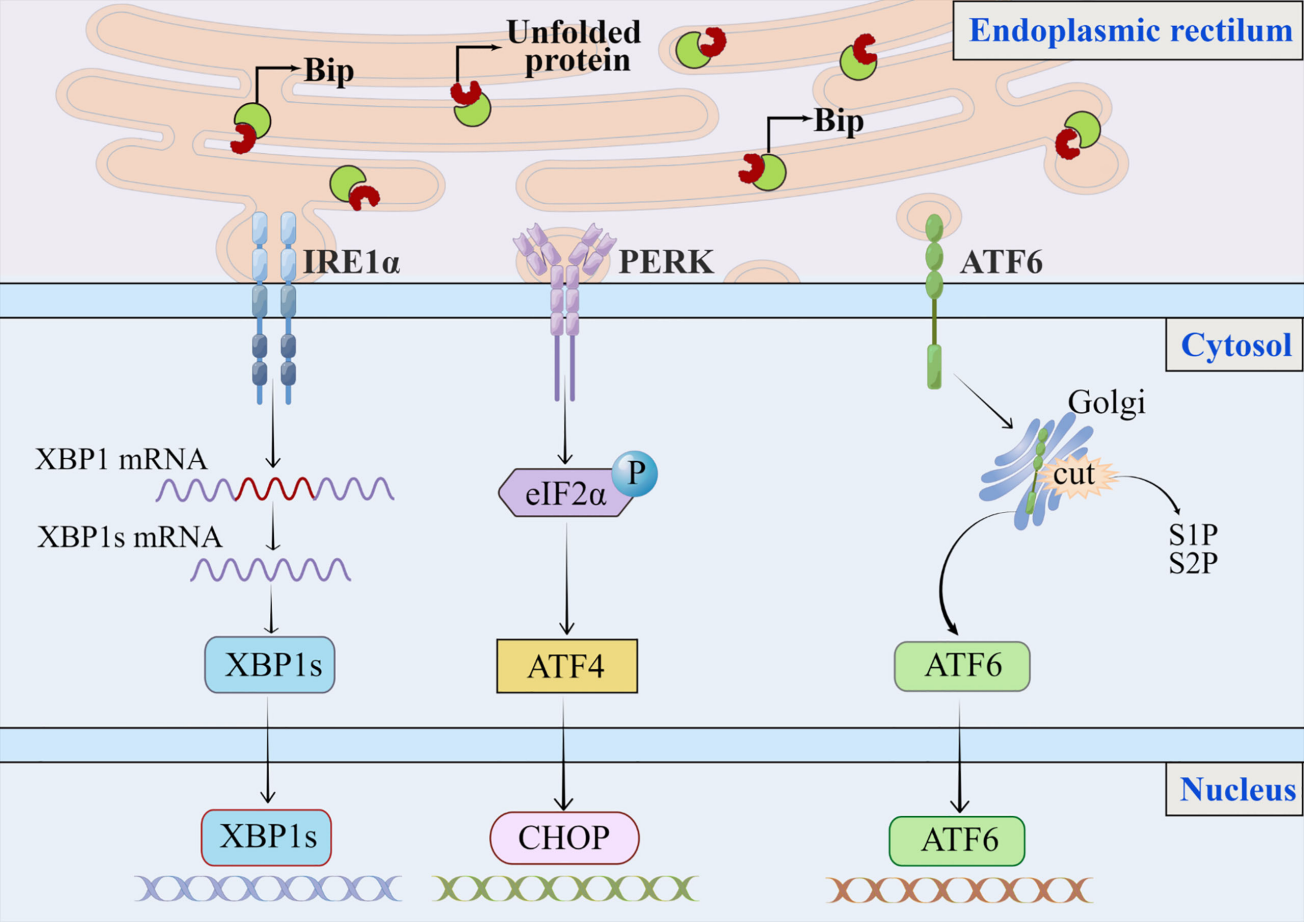
The classic endoplasmic reticulum stress signaling pathway. In response to stress or physiological conditions, the endoplasmic reticulum molecular chaperone GRP78/Bip binds to three transmembrane ER resident pressure sensors (A) PERK, (B) IRE1, and (C) ATF6. When endoplasmic reticulum stress occurs, misfolded or unfolded proteins accumulate in the lumen of the ER, GRP78/Bip dissociates from these three pressure sensors and binds to misfolded or unfolded proteins, triggering the UPR. The extent to which ER stress and the UPR contribute to the NAFLD process may depend on the ability of the UPR to mitigate the damage that leads to disrupted ER homeostasis. (A) PERK phosphorylates eIF2α. To alleviate protein overload in the ER, phosphorylation of eIF2α reduces translation of mRNAs but can increase translation of some specific mRNAs, such as ATF4.(B) Accumulation of unfolded protein in ER induces oligomerization of IRE1α on ER membrane and autophosphorylation of IRE1α cytoplasmic structural domain, and autophosphorylation of IRE1α can further activate ribonuclease activity; and IRE1 has endonuclease activity, which will splice XBP1 mRNA into XBP1s, encoding transcription factors and activating expression of UPR target genes.(C) ATF6 moves as a vesicle from the ER to the Golgi apparatus, where it is cleaved by S1P and S2P then migrates to the nucleus to activate XBP1 and genes involved in ER protein folding and secretion, such as CHOP. ER, endoplasmic reticulum; UPR, unfolded protein response; S1P, site 1 protease; S2P, site 2 protease.

The purpose of UPR is to maintain hepatic physiology by protecting hepatocytes from cellular stress due to increased secretory demand or cellular differentiation (13). While under physiological conditions, the liver experiences transient ER stress and quickly returns to normal. In chronic diseases such as NAFLD, this stress may become chronic and then promote the progressions to a more severe stage, such as liver cirrhosis or HCC, by inducing inflammatory responses and cell death (14, 15). The induction of UPR involves the activation of three transmembrane ER resident stress sensors: PERK-eIF2α-ATF4(RNA dependent protein kinase-like ER kinase—the eukaryotic translation initiation factor eIF2α—activating transcription factor 4), IRE1-XBP1(inositol-requiring enzyme 1—X box binding protein-1), and ATF6 (activating transcription factor 6) (13, 14), which aim to increase protein folding capacity by reducing protein translation to restore ER homeostasis and promote degradation of misfolded or unfolded proteins **(** **Figure 1** **) (** 8). When hepatocytes are in non-stressed or physiological conditions, these proteins remain inactive and bind to the molecular chaperone GRP78/Bip (glucose-regulated protein 78/binding immunoglobulin protein), which is also known as a major regulator of ER stress (13, 16). GRP78 disintegrates from these three stress sensors following intracellular ER stress, leading to their activation. The extent to which ER stress and the UPR contribute to the NAFLD disease process may depend on the ability of the UPR to mitigate the damage that leads to disruption of ER homeostasis.

### PERK-eIF2α-ATF4 pathway

The PERK-eIF2α-ATF4 pathway leads to the up- regulation of UPR target genes and induces the proapoptotic protein C/EBP homologous protein (CHOP), regulating both lipogenesis and hepatic steatosis. PERK, PKRlike endoplasmic reticulum kinase, also known as eukaryotic translation initiation factor 2αkinase (eIF2α) 3, also contributes to hepatic stellate cells (HSC) activation (17). To alleviate protein overload in the ER, phosphorylated eIF2α blocks mRNA translation by preventing the assembly of 80s ribosomes, while paradoxically increasing the translation of several mRNAs with upstream open reading frames in the 5’ region, such as ATF4 (18). Prolonged ER stress can induce autophagy mediated by PERK through ATF4, increasing expression of key autophagy-related proteins necessary for autophagosome formation (7). Protein kinase mediated phosphorylation of eIF2α increases the translation of ATF4, and eIF2α phosphorylation can greatly reduce the functional load of the ER by reducing the synthesis of new proteins that need to be folded. It was shown that ATF4 gene knockout mice were protected against diet-induced obesity, hyperlipidemia, and hepatic steatosis. In addition, ATF4 deficiency significantly reduced the expression of lipogenic nuclear receptor peroxisome proliferator-activated receptor (PPARγ), sterol regulatory element binding protein (SREBP1c), acetyl coenzyme A carboxylase and fatty acid synthase in liver and white adipose tissue (19–21). Another study has confirmed that ER stress reduces apolipoprotein B 100 (ApoB100) by degrading ApoB100 and impairing ApoB100 translation through the PERK-ATF4 branch of the UPR. ApoB100 is one of the apolipoproteins of very low-density lipoprotein (VLDL) and low-density lipoprotein (LDL), both are rich in cholesterol and whose main role is to transport cholesterol into the peripheral circulation (22). The decrease in ApoB100 caused by the PERK-ATF4 branch increases blood cholesterol levels, causing liver steatosis. Pre-clinical studies have shown that carbon monoxide upregulates sestrin-2 through the PERK-eIF2α-ATF4 signaling pathway and alleviates dietary methionine/choline deficiency induced hepatic steatosis (23). Salubrinal is a selective inhibitor of eIF2α dephosphorylation, which maintains the phosphorylation state of eIF2α and protects cells from ER stress-induced apoptosis (24). By inhibiting the dephosphorylation of eIF2α in ER stress, Salubrinal reduces hepatic steatosis and fat deposition (25).

### IRE1α-XBP1 pathway

Inositol-requiring protein 1 (IRE1α) is a type I bifunctional transmembrane protein with serine/threonine protein kinase and endonuclease activities, and the accumulation of unfolded proteins in the ER induces oligomerization of IRE1α on the ER membrane and autophosphorylation of IRE1α cytoplasmic structural domain (26), the autophosphorylation of IRE1α can further activate ribonuclease activity. Activated IRE1α processes an intron of X box binding protein-1 (XBP-1) mRNA, leading to unconventional splicing, followed by mRNA rejoining and eventual translation to produce active transcription factors XBP1s; XBP-1 binds to the promoters of several genes involved in UPR and ER-associated degradation (ERAD) in order to maintain ER dynamic homeostasis and prevent cytotoxicity (27), and XBP1s enhance ER protein folding, secretion, ERAD and lipid synthesis (28). Activated IRE1α also recruits tumor necrosis factor receptor (TNFR)-related factor 2 (TRAF2) and apoptosis-signaling kinase 1 (ASK1) to mediate activation of c-jun amino-terminal stress kinase (JNK) and nuclear factor kappa B (NF-κB) (29, 30). Mice with hepatocyte-specific deletion of IRE1α exhibit increased hepatic steatosis and decreased plasma lipids under ER stress conditions due to altered expression of key metabolic factors such as C/EBPβ, C/EBPδ, PPARγ, and enzymes involved in triglyceride biosynthesis (31), and IRE1α is also required for the efficient synthesis of ApoB (32). This suggests that the transactivator protein IRE1α in the UPR inhibits lipid accumulation in the liver, especially under ER stress conditions. Although IRE1α is protective, it blocks basal levels of UPR in the liver, which may lead to increased ER stress (14). XBP1 expression is significantly upregulated in liver samples from patients with NASH, and inhibition of the XBP1 signal significantly reduced serum triglyceride, cholesterol and fatty acid levels by reducing the metabolism of liver lipogenesis in mice (33). Inhibition of the IRE1α pathway in HSC can reduce both their activation and autophagic activity, resulting in a reduced fibrogenic response (34). Therefore, XBP1 inhibition may prevent steatohepatitis, and XBP1 is a potential therapeutic target for NASH (33).

### ATF6 pathway

ATF6 is a type II transmembrane protein on the ER membrane and is distributed as a proenzyme in the non-stressed state; in ER stress, ATF6 is metastasized to the Golgi apparatus in the form of the vesicle (35, 36). In the Golgi apparatus, both ATF6 and SREBPs are activated by the same proteases site-1 protease and site-2 protease (37, 38), which then migrate to the nucleus under the pull of nuclear localization signals (38) to induce transcriptional expression of ER stress genes, including CHOP/XBP-1 in the nucleus. Studies of ATF6 activity and SREBP2-mediated lipogenesis indicate that ATF6 overexpression binds to and inhibits transcription and lipogenesis accumulation of SREBP2 regulated lipogenic genes (39), but this inhibition can be reversed by blocking ATF6 cleavage by GRP78/BiP (40). Researchers have shown that ATF6 plays a “dual role” in the development of diabetes. On the one hand, ATF6 protects β cells from ER stress, inhibits hepatic steatosis, and reduces hyperglycemia and hyperinsulinemia in obese mice with hepatic overexpression (41); on the other hand, ATF6 is also involved in the development of hyperlipidemia and insulin resistance (42). Deficiency in ATF6 prevents steatosis during chronic ER stress, but exacerbates it during acute ER stress, suggesting that ATF6 plays both a protective and a pathological role in fatty liver (43). Recent studies have shown that the activation of the ATF6 signaling pathway can promote the progression of NAFLD, and the down-regulation of the pathway can inhibit the disease progression by reducing ER stress-induced inflammation and hepatocyte apoptosis (44).

Generally, under ER stress, Bip binding to unfolded proteins dissociates the tubular domain of the sensor, which then leads to activation of IRE1α and PERK through transphosphorylation and ATF6α through a protein hydrolysis process (45, 46). ATF6 enhances XBP1 mRNA expression, providing additional substrate for IRE1α to splice into a more transcriptionally active form; whereas the unspliced XBP1 protein is intracellularly unstable and can heterodimerize with ATF6 and sXBP1, which promotes their proteasomal degradation (47, 48). Upon activation of the three pathways, the UPR signaling pathway induces the expression of genes encoding functions that improve the stress state of the ER.

## The role of innate immune cells and ER stress in NASH

**Figure 2:**
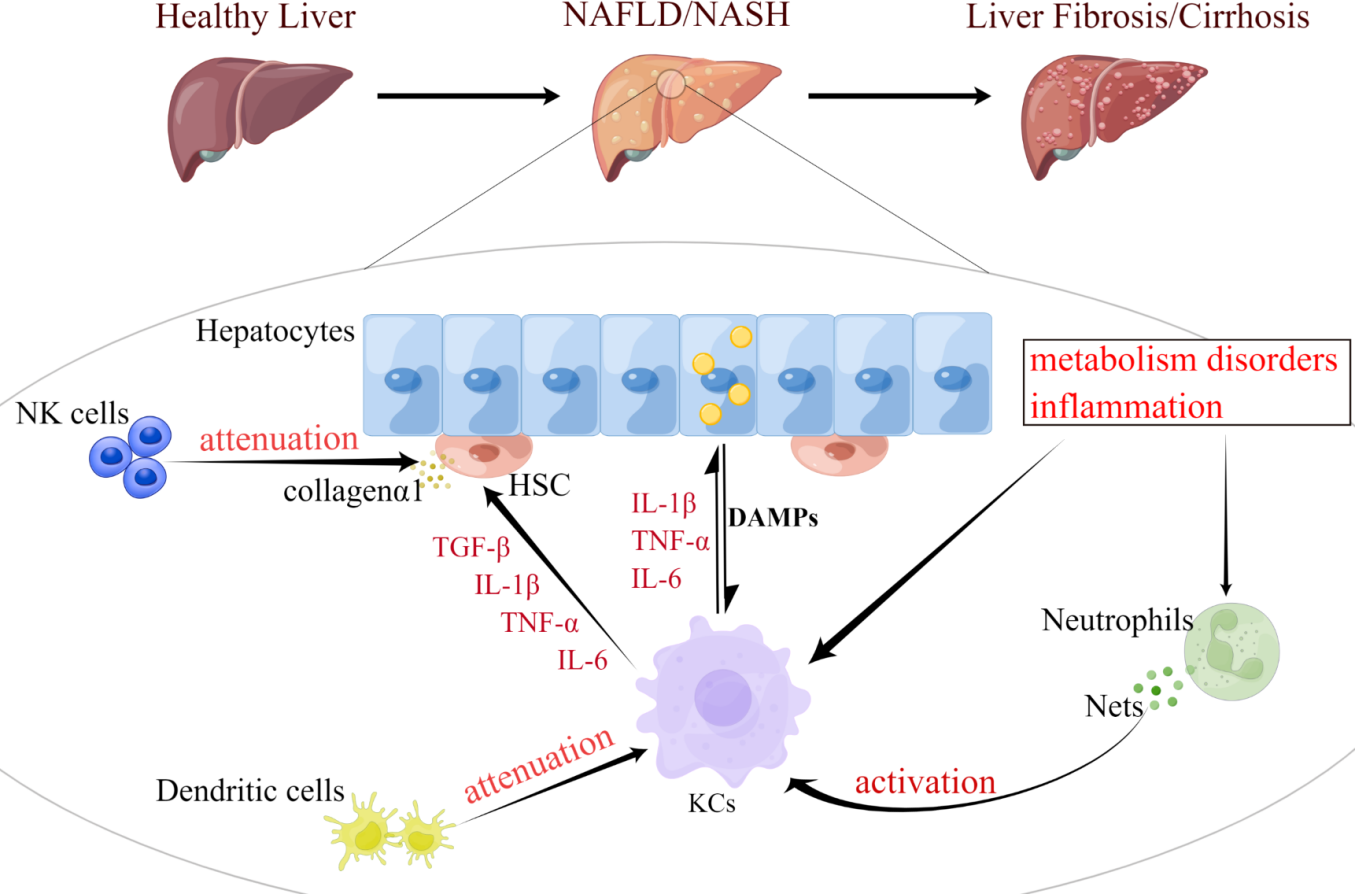
Functional changes of innate immune cells are involved in the progression of NAFLD. NAFLD progression occurs in parallel with metabolic and inflammatory derangements that promote the activation and aggregation of innate immune cells (e.g., KCs, neutrophils, DCs, and NK cells). During the development of NASH, KCs can be activated by excessive fat load in hepatocytes, dysregulated hepatic metabolism or inflammation. Fat overload in hepatocytes induces the release of lipotoxic and DAMP, activating KCs and HSC, thereby promoting inflammation and fibrosis. Neutrophils induce metabolic inflammation in the liver by releasing high levels of granulins, forming NETs, and activating KCs. DCs can also activate KCs and activated KCs can exacerbate hepatocyte steatosis by secreting cytokines, such as IL-1β, TNF-α, and IL-6. Meanwhile, both KCs and NK cells promote the activation and survival of HSC, which trigger their release of collagen 1, as well as the development of liver inflammation and fibrosis. KCs, Kupffer cell; DCs, dendritic cell; HSC, hepatic stellate cell; NK cell, natural killer cell; DAMP, damage-associated molecular patterns; NETs, neutrophil extracellular traps; IL-1β, interleukin 1 beta; TNF-α, tumor necrosis factor alpha.

Activation of innate immunity further drives the infiltration and accumulation of inflammatory cells in the liver, thereby exacerbating inflammation and injury (49). Pro-inflammatory mediators produced by immune cells and their damage trigger activation of HSC involved in fibrosis. Innate immune cells such as neutrophils or macrophages are the central regulatory cells of NASH-related inflammation **(** **Figure 2** **)**. Macrophages are crucial in driving this process. Other Immune cells, such as T cells, cytokines, death ligands and oxidative stress may also promote hepatic stellate cell apoptosis. Senescent cells are subsequently eliminated by NK cells. Given the central role of innate immunity in NAFLD pathogenesis, this section discusses recent advancements in the function of innate cell subsets and the effects of ER stress in NAFLD and NASH.

### Macrophage

Macrophages are key components of the innate immune system and in the liver include liver-resident Kupffer cells (KCs) and recruited circulating monocyte-derived macrophages (50–52), which constitute the largest natural immune cell population in the liver. Hepatocyte fat overload induces the release of lipotoxic and damage-associated molecular patterns (DAMP), activating KCs and hepatic stellate cells HSC, which respectively promote inflammation and fibrosis (53); and activated KCs then produce inflammatory cytokines and chemokines, such as tumor necrosis factor-α (TNF-α), interleukin-1β (IL-1β), and leukocyte interleukin-6 (IL-6), to induce hepatocyte injury and inflammatory necrosis (49). Macrophages are activated and polarized by metabolic changes that allow them to adapt to microenvironmental changes associated with inflammation or tissue damage (hypoxia, nutritional imbalance, oxidative stress, etc.) and to perform their highly energetic pro-inflammatory and antimicrobial function (54, 55). For example, during inflammation, KCs infiltrate into the liver and participate in the progression of various liver diseases; the phenotype and function of monocyte derived hepatic macrophages are highly dependent on local stimulation during liver disease and both together play a key role in the regulation of inflammation, fibrosis and fibrosis (56, 57). RNA sequence analysis showed that both KCs and monocyte derived macrophages upregulated the expression of inflammatory cytokines, whereas monocyte derived macrophages were more likely to express growth factors associated with angiogenesis and liver fibrosis (58). In the early stages of liver injury KCs play a crucial role by producing tumor necrosis factors and chemical inducers that trigger the recruitment of circulating monocyte-derived macrophages, rapidly acquiring a pro-inflammatory phenotype and amplifying the development of NASH and liver fibrosis (59). In response to liver injury, KCs recruit blood immune cells and then differentiate into CD11b^+^F4/80^+^ classically activated macrophages (M1 type) with phagocytic activity and secretion of pro-inflammatory cytokines and reactive oxygen species (ROS); M2 type macrophages induce M1 type macrophage apoptosis *in vitro* through IL-10 paracrine activation of arginase (60). Mitochondrial DNA in high-fat diet (HFD)-fed mouse hepatocytes activates KCs and induces cytokine release, steatosis, and inflammation through the interferon gene stimulator (STING) pathway (61). According to a study conducted on children with NAFLD, activated macrophages were located in the interstitial space between damaged hepatocytes. When NASH occurs, high levels of endotoxin induced by increased intestinal permeability and/or danger signals from lipotoxic hepatocytes stimulate KCs to produce transforming growth factor (TGF)-β, IL-1β, and TNF-α. Then the inflammatory factors stimulate HSC, they can mediate immunoregulatory effects by functioning as non-professional antigen presenting cells in the injured liver. As the same time, they increase hepatic collagen-α1 production to ultimately trigger fibrosis (59). Therefore, NASH facilitates infiltration of pro-inflammatory macrophages and promote the activation of HSC, which conversely increases liver injury, inflammation and fibrosis, creating a vicious cycle (62).

**Figure 3:**
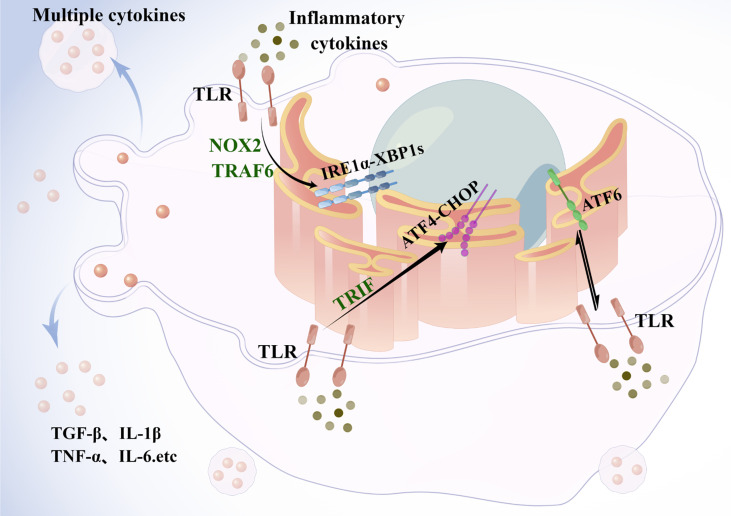
Endoplasmic reticulum stress in Kupffer cells. Under metabolic and inflammatory conditions, the UPR signaling pathway is activated in the ER. In macrophages, TLR signaling pathway induces ER stress, and TLR2 and TLR4 induce activation of IRE1α, followed by activation of xbp1, through a mechanism that requires the NADPH oxidase NOX2 and TRAF6. ATF6, by mediating a proinflammatory synergy between ER stress and TLR activation is involved in the development of liver injury. The TLR signaling pathway adaptively inhibits the ATF4-CHOP branch of the UPR in a TRIF-dependent manner. Activated KCs then release cytokines such as TNF to act synergistically with other immune cells to exacerbate hepatic steatosis and fibrosis. TLR, toll-like receptor; TRAF6, TNF receptor-associated 6; TRIF, TIR structural domain-containing adapter-induced interferon-β.

And the ER stress response is critical for the integration of metabolic and inflammatory responses in KCs **(** **Figure 3** **)**. Under conditions of metabolism and inflammation, the UPR signaling pathway in the ER is activated. In KCs, toll-like receptor(TLR) signaling induces ER stress, which triggers the TLR response upon binding to its ligand (63). TLR2 and TLR4 induce IRE1α activation through a mechanism that requires NADPH oxidase NOX2 and TNF receptor-associated 6 (TRAF6), and subsequently induce XBP1s activation (64). Similarly, ATF6 contributes to the pathogenesis of liver ischemia-reperfusion injury through meditating a pro-inflammatory synergy between ER stress and TLR activation (65). On the other hand, ATF4 links metabolic stress to IL-6 expression in macrophages (66), while the TLR signaling pathway adaptively inhibits the ATF4-CHOP branch of the UPR in a TRIF (TIR structural domain-containing adapter-induced interferon-β)-dependent manner (67). In an experimental model of lung injury and fibrosis, CHOP deficiency in mice promotes macrophage accumulation by inhibiting ER stress-induced cell death. The results indicate that GRP78 inhibits pulmonary fibrosis, while CHOP upregulation promotes pulmonary fibrosis (68, 69). Therefore, macrophages, either liver-resident KCs or circulating monocyte-derived macrophages, have great phenotypic plasticity, and they may positively or negatively influence the development of NASH.

### Neutrophil

The neutrophil is the most abundant white blood cell in human blood and the primary player in the innate immune response (70). There are virtually no resident neutrophils in the liver, but when the liver undergoes pathogens invasion, acute inflammation or injury, neutrophils are the first to reach the lesion and integrate chemotactic signals into a migratory response toward tissue injury (71, 72). In the presence of IFN-β, IL-1β, IL-8, and TNF-α, neutrophils polarize toward N1 (73). N1 neutrophils are characterized by short lifespan, mature phenotype, high cytotoxicity, high immune activity, and promotion of CD8^+^ T cell activation (74, 75). Experimental data suggests that CD8+ T cells could play a pro-fibrogenic role in the liver. However, IFN-γ can change the phenotype of hepatic CD8^+^ T cells towards increased cytotoxicity and its absence attenuated liver fibrosis in chronic sclerosing cholangitis (76). In the effect of TGF-β, IL-8, IL-6, and IL-17, neutrophils polarize toward N2, which has a long lifespan, immature phenotype, low cytotoxicity, and promotes tumor growth, infiltration and metastasis (77). Complex mechanisms help neutrophils get involved in immunity and inflammation, including phagocytosis, superoxide production, cytokine and chemokine production, degranulation and the formation of neutrophil extracellular traps (NETs) (71, 78). These mechanisms play an important role in acute aseptic liver injury, however, their role in metabolism-induced chronic liver disease in NAFLD requires further investigation. Recently, NETs show a facilitative role in NAFLD progression. In the serum of NASH patients, the levels of myeloperoxidase (MPO)-DNA complexes elevate, which are NET biomarkers, have been found (79, 80). In addition, neutrophil infiltration into the liver of NASH mice and promotion of NETs formation, and the synergy of the two can promote the development of NAFLD into hepatocellular carcinoma in mice (80). Inhibition of NETs formation by deoxyribonuclease (Dnase) treatment or by using peptide arginine deaminase type IV-deficient (PAD4^-/-^) mice significantly reduced macrophage infiltration, inflammatory cytokine production, and the progression of NASH to hepatocellular carcinoma (81). Some studies show that neutrophil elastase (NE)/α1-antitrypsin ratio, plasma proteinase 3 (PR3) and NE concentrations (82), neutrophil/lymphocyte ratio (83), NETs levels and MPO levels (81) are significantly elevated in patients with NAFLD. In short, neutrophils promote metabolic inflammation in the liver through releasing high levels of granule proteins, as well as forming NETs and interacting with other pro-inflammatory immune cells.

During neutrophil differentiation, the activity of PERK and ATF6 decreases and the activity of IRE1α increases, activation of the IRE1α-XBP1 pathway is the basis of neutrophil differentiation (84). Traditionally, apoptosis of neutrophils is mainly activated by endogenous and exogenous pathways. However, several key molecules of the UPR, such as GRP78, ATF6, XBP1 and eIF2α, are found to be highly expressed in neutrophils treated with arsenic trioxide ATO or other ER stress-inducing inducers. These results suggest that the ER stress-mediated apoptotic pathway plays a role in human neutrophils (85). Several studies show that human NE can induce apoptosis in endothelial cells by activating the PERK-CHOP branch of the unfolded protein response (86). In lupus disease, neutrophils amplify inflammation in the disease by releasing NETs, and elevated the ER stress sensor IRE1α activity associated with overall disease activity can be detected in neutrophils isolated from lupus patients, suggesting that the ER stress sensor IRE1α drives neutrophil hyperactivity in lupus (87). Thus, UPR is important for both neutrophil stage-specific and intensity-specific differentiation by reducing ER stress during neutrophil differentiation, maintaining UPR and controlling ER stress (88). After neutrophils infiltrate the liver, either by their differentiation or apoptosis, it is not difficult to speculate that they are regulated by the UPR, which in turn regulates the occurrence of their ER stress. When the balance is disturbed, ER stress in neutrophils promotes disease progression.

### Dendritic cell

Dendritic cells (DCs), which originate from bone marrow pluripotent hematopoietic stem cells, are the most functional and specialized antigen presenting cells (APC) in the body, acting as a cellular connector between innate and adaptive immunity. DCs can efficiently uptake, process and present antigens (89). DCs migrate from the blood to the lymph nodes through the hepatic sinusoids, so the hepatic sinusoids can serve as an important enrichment area for hepatic DCs (90). Hepatic dendritic cells (HDCs) are a heterogeneous group of bone marrow-derived cells involved in the regulation of antigen presentation to lymphocytes and the hepatic immune response (51, 91, 92). HDCs are mainly localized in the portal area and can be classified according to the expression of specific markers: plasmacytoid-like dendritic cells (PDCA-1^+^; pHDCs); myeloid or classical dendritic cells (PDCA-1^-^; cHDCs/mHDCs), the latter were further subdivided into CD103^+^/CD11b^-^ type 1 (mHDC1) and CD103^-^/CD11b^+^ type 2 (mHDC2) cells (91, 92). pHDCs secrete type I interferons (IFNs) during viral infection, whereas cHDCs present antigens to T cells (93). When a liver injury occurs, mHDCs proliferate and activate as efficient antigen-presenting cells, producing large amounts of pro-inflammatory cytokines (94). However, it has been found that type I myeloid HDCs (CD103^+^/mHDC1) have an anti-inflammatory ability, affecting the conversion from steatosis to steatohepatitis, and it has been suggested that different subsets of mHDCs may have opposite effects in regulating lobular inflammation in human NAFLD/NASH (95). Therefore, the role of HDCs in the progression of NAFLD disease needs further study.

Three pathways of UPR are involved in the *in vivo* homeostasis and control of immune responses in DCs (96, 97). The PERK-CHOP branch increases IL-23 expression in human DCs upon LPS and tunicamycin stimulation (98), which is a cytokine associated with protective immunity against some pathogens (99). In cancer, the IRE1α-XBP1 pathway can active DCs of the tumor microenvironment and regulates antitumor immunity to evade immune control (100–102). During acute inflammation, elevated fatty acids (FA) production from lipolysis in adipose tissue may enhance the production of IL-23 and IL-6 by DCs, thereby promoting inflammatory effects against pathogens. Excessive FA during obesity and HFD feeding may lead to excessive activation of UPR in DCs, exacerbating inflammation through DC-specific XBP1-dependent regulation of IL-23 production and promoting DCs differentiation by enhancing TLR signaling to stimulate inflammatory cytokine gene production and late metabolic adaptation of TLR-activated DCs to a high FA environment leading to synergistic induction of UPR (103). And XBP1 plays a key role in reducing the immunogenicity of DCs by promoting the synthesis and accumulation of fatty acids and triacylglycerols (103).

### Natural killer cell and natural killer T cell

Natural killer cells (NK cells) belong to the innate lymphoid cell family and are involved in early defense against foreign cells, as well as experiencing various forms of stress. IRE1α and its substrate XBP1 drive NK cells response to viral infection and *in vivo* tumor, as well as being critical for the proliferation of activated mouse and human NK cells (104). NK cells usually exhibit anti-fibrotic properties, including killing activated HSC by secreting interferon gamma, and also help to clear senescent activated hepatic stellate cells (76, 105).The functions of NK cells are strongly regulated by the stimulation of multiple surface-activated and inhibited receptors. Various studies show that NK cells activation in NASH may be associated with elevated levels of several NK cell-activating cytokines, such as IL-2, IL-12 and IFN-α/β (106). However, there are discrepant data in this regard as obese subjects with NAFLD and rats fed with a diet deficient in methionine and choline (MCD), which induces NASH, exhibit decreased cytotoxic activity of NK cells.

Natural killer T (NKT) cells comprise a unique immune cell subtype that expresses specific NK cell surface receptors as well as an antigen receptor (TCR) characteristic of conventional T cells. Similar to NK cells, NKT cells have antifibrotic effects by directly killing activated HSC (107). However, another study suggests NKT cells can also accumulate in progressive NASH, thereby promoting the fibrotic process. Depletion of these cells resulted in reduced NASH progression and thus presents novel therapeutic avenues for the treatment of NASH (108, 109). In mice fed with a high fat or sucrose diet, increased apoptosis of NKT cells was induced in the liver, which resulted in the reduced NKT cells and promoted hepatic inflammation by excessive production of IFN-γ and TNF-α (110). The classification may play a significant role in these differences. Studies have pointed out that there are at least two NKT cells subsets, which play opposite roles in liver inflammation. Type I NKT cells is pro-inflammatory, while Type II NKT cells has protective effects on liver injury (111). Interestingly, type I NKT cells are easily activated by lipids and therefore may play a role in NAFLD.

## Treatment

The ideal therapy would effectively reverse the lipid accumulation, liver inflammation, liver injury and fibrosis, although a wealth of information on the pathogenesis of NASH has accumulated during the past 10 years, there are no specific therapeutic drugs for NAFLD/NASH. Cholesterol­lowering drugs such as ezetimibe or statins can reverse hepatic free cholesterol accumulation and attenuate steatohepatitis and fibrosis in a mouse model of NASH (112), but their activity in humans has not yet been rigorously assessed in large numbers of patients. Currently, what is clear is that both genetic and lifestyle factors play a non-negligible role in the development of NAFLD. Lifestyle changes, such as improved diet, weight management and increased physical activity, are effective strategies to prevent and treat NAFLD (113, 114). These measures aim to eradicate NASH and other diseases related to metabolic syndrome. A prospective cohort study of paired liver biopsies in 261 patients suggested that weight loss of more than 5% may be associated with fibrosis stabilization and regression (115). Many current pharmacological approaches to the treatment of NASH focus on events such as liver injury, inflammation and fibrosis (**Table 1**).

**Table 1:** Therapies for non-alcoholic steatohepatitis (NASH).

Drug	Mechanism of action	Functions	
*Effects on lipid metabolism*			
**Salubrinal**	⁃Selective inhibition of eIF2α dephosphorylation (24) ⁃Inhibition of ER stress and reminder of autophagy through eIF2α signaling (25)	⁃Attenuates hepatic steatosis and fat deposition	
**Obeticholic acid**	⁃An agonist of the FXR (116)	⁃Decreases hepatic lipogenesis, steatosis, and insulin resistance ⁃Inhibits inflammatory and fibrogenic responses in NASH patients	
**Rapamycin (** 117)	⁃Selectively inhibition of mTOR ⁃Inhibition of ER stress		⁃Improves hepatic steatosis
**Matrine**	⁃Competitive inhibition of the SERCA (118)		⁃Improves the ER stress state to reduces lipid metabolism disorders, mitochondrial dysfunction and inflammatory responses
**Empagliflozin**	⁃Reduced expression of adipogenic genes and endoplasmic reticulum stress-related genes (119)		⁃Reduces adipogenesis and endoplasmic reticulum stress (120)
**Vitamin E (** 121)	⁃An antioxidant ⁃Inhibiting the late maturation of SREBP-1c to reduce hepatic new lipogenesis		⁃Mediates the reduction of hepatic new lipogenesis (122) ⁃Improves lobular inflammation and no increase in fibrosis
**Liraglutide (** 123)	⁃A synthetic long-acting glucagon-like peptide 1 (GLP-1) receptor agonist		⁃Be effective in weight loss, resolution of steatohepatitis and less progression of fibrosis (124)
*Other treatments*			
**Sivelestat**	⁃An inhibitor of neutrophil elastase		⁃Inhibits the infiltration and activation of neutrophils and apoptosis and reduces proinflammatory factor

### Effects on lipid metabolism

As mentioned earlier, a possible mechanism by which Salubrinal attenuates hepatic steatosis and fat deposition is by inhibiting ER stress and alerting autophagy *via* eIF2α signaling (25). The bile acid receptor farnesoid X receptor (FXR) is a member of the nuclear hormone receptor superfamily that is highly expressed in the liver (116). FXR ligands have many beneficial effects treating NAFLD and/or NASH by decreasing hepatic lipogenesis, steatosis, and insulin resistance while also inhibiting inflammatory and fibrogenic responses in NASH patients (125–127). Obeticholic acid (OCA) is an agonist of FXR, OCA reduces endogenous bile acid production by down-regulating SREPB-1C, which helps to improve the histological features of NASH (128). Rapamycin improves hepatic steatosis by selectively inhibiting mammalian target of rapamycin (mTOR) and inhibiting ER stress (117). Matrine, a competitive inhibitor of the SarcoEndoplasmic Reticulum Calcium ATPase (SERCA), improves the ER stress state, which reduces lipid metabolism disorders, mitochondrial dysfunction and inflammatory responses (118). Vitamin E, which mediates the reduction of hepatic new lipogenesis by inhibiting the late maturation of SREBP-1c (122). According to a clinical study, in NAFLD, compared with placebo, vitamin E therapy demonstrated improvement in steatosis or lobular inflammation and no increase in fibrosis (121). However, the long-term safety of vitamin E is controversial due to its potential risk for increased mortality (129). In mice treated with empagliflozin, according to protein expression, the expression of PPARα was higher in the experimental group, and the expression of lipogenic genes SREBP-1c and PPARγ was concomitantly reduced, along with a decrease in genes associated with ER stress CHOP, ATF4 and GADD45 (119). Therefore, it is not difficult to speculate that empagliflozin reduces adipogenesis and ER stress by suggesting that empagliflozin may be an important tool in the treatment of progressive hepatic steatosis. A small phase 2 trial that assessed the safety and efficacy of liraglutide, a synthetic long-acting glucagon-like peptide 1 (GLP-1) receptor agonist currently available for the treatment of type 2 diabetes and obesity, in patients with NASH found the drug to be effective in weight loss, resolution of steatohepatitis and less progression of fibrosis in patients with NASH, but further studies are needed (124).

### Other treatments

Broad spectrum antibiotics reduce bacterial translocation and TLR4-dependent macrophage activation to alleviate steatohepatitis and fibrosis in mice (130). Thus, affecting the gut microbiota through probiotics, antibiotics, and modifying bile acid composition may potentially mitigate the activation of pathogenic Kupffer cells in the liver (131). In liver fibrosis, studies indicate that a cell therapy approach (for example, the delivery of bone marrow-derived macrophages) could potentially induce pro-regenerative effects (132). On the other hand, NE inhibitor sivelestat treatment inhibits the infiltration and activation of neutrophils and apoptosis and reduces pro-inflammatory factors such as TNF-α and IL-6, and downregulates chemokines (133).

The current treatment for NAFLD/NASH is limited to lifestyle modifications, and no drugs are currently officially approved as treatments for NASH. Therefore, it is necessary for us to pursue the development of medications for the treatment of NASH. Given the multiple pathways implicated in NASH pathogenesis and observed response from single-agent therapies, combination and individualized regimens will likely be needed to adequately treat NASH. However, there is little targeted treatment available, and liver transplantation remains the only potentially effective treatment available, so controlling disease progression in the early stages of the disease (whether it is alcoholic liver disease or NASH, etc.) through interventions such as inflammation is a more effective treatment.

## Conclusion and perspective

Significant advances in understanding the history and underlying mechanisms of NAFLD development in the past decades. In recent years, due to the in-depth understanding of the pathogenesis of NAFLD and the increasing prevalence of NAFLD, the diagnosis of NAFLD requires a “positive standard”. Therefore, in 2020, NAFLD was proposed to be replaced by MAFLD (134, 135). This is a consensus statement issued by an international panel of 30 experts from 22 countries that provides a comprehensive and simple diagnosis of MAFLD and can be applied to any clinical setting (135). This name change is the result of 40 years of research and understanding with a new milestone significance. The new diagnostic criteria for MAFLD are based on the presence of fatty liver indicated by liver biopsy histology or imaging or even blood biomarker examination, and meeting one of the following three conditions: overweight/obesity, type 2 diabetes, or metabolic dysfunction (135). This update of nomenclature will be a step towards further characterizing the pathology of the disease. Previous studies suggest that ER stress can aggravate lipid accumulation in the liver by increasing the synthesis of fatty acids, and activation of the IRE1α pathway may lead to hepatic insulin resistance accelerating the development of MAFLD; additionally, it can increase the expression of inflammatory factors, which may contribute to the development of NASH. This mechanism is particularly obvious in MAFLD caused by high fructose and has been validated by experimental treatment (136).

In animal models and clinical studies, innate immunity cells have been demonstrated to play a crucial role in the development, propagation, as well as modulation and amelioration of liver inflammation as it pertains to NASH. It is clear that innate immunity contributes to liver immune cell infiltration, further aggravating liver damage and inflammation. As a consequence of this inflammatory process, HSC is activated, which later promotes inflammation and liver fibrosis, ultimately promoting the development of cirrhosis. It is estimated that as many as 7 million of the total population of China have cirrhosis of the liver, with 460,000 new cases of liver cancer occurring each year (137). Compared with healthy individuals, patients with compensatory and decompensated cirrhosis had five-fold and 10-fold increases in mortality risks, respectively (138). Portal hypertension occurs in decompensated cirrhosis, and decompensated events such as ascites, hepatic encephalopathy, bleeding esophagogastric fundic varices and hepatorenal syndrome may occur, which arise in the context of cirrhosis-related immune dysfunction and determine morbidity and prognosis (139). Targeting strategies should be disease-specific, either to enhance, inhibit or restore the function of immune cells, and some strategies are already in clinical use or different clinical trial phases (140). Macrophages and other immune cells in liver play an important role in triggering and amplifying liver inflammation and fibrosis in NAFLD/NASH, and it is not difficult to imagine their impact on NAFLD/NASH after the occurrence of ER stress. Therefore, there is great potential for research on drugs targeting immune cells and their ER stress, myeloid cells and products may represent potential therapeutic targets and noninvasive markers of disease severity.

However, there are still many challenges left to overcome. Researchers increasingly understand the importance of addressing the risk factors of NAFLD from a multi-pronged public health approach due to the scarcity of awareness in the general population and treatments for such diseases. Furthermore, new techniques such as single-cell RNA sequencing, multiparameter histological analyses or multiple paired liver biopsies will help overcome some of these challenges. In conclusion, early identification and targeted treatment of patients with nonalcoholic steatohepatitis can greatly assist in improving patient prognosis, including guiding patients to intensive lifestyle modifications to promote weight loss and referral to bariatric surgery, as indicated by the management of obesity and metabolic diseases. It is believed that our in-depth study of the inflammatory immune microenvironment of the liver will provide a more effective treatment for inflammation and fibrosis caused by the progression of NAFLD. In the future we need more efforts to explore the targeting of therapies, whose successful application will require an unprecedented interdisciplinary approach, which will obviously be a multidisciplinary combination of molecular biology, immunology, pharmacology, genetics, chemistry and technological advances in nanotechnology.

## Author Contributions

LZ, HS, and XL contributed to conception and design of the work. LZ wrote the first draft of the manuscript. HS and HW helped in revision and edited the article before submission. All authors contributed to the article and approved the submitted version.

## Funding

This work was supported by grants from the National Natural Science Foundation of China (U21A20375), and the Postgraduate Innovation Research and Practice Program of Anhui Medical University (YJS20210274).

## Conflict of Interest

The authors declare that the research was conducted in the absence of any commercial or financial relationships that could be construed as a potential conflict of interest.

## Publisher’s Note

All claims expressed in this article are solely those of the authors and do not necessarily represent those of their affiliated organizations, or those of the publisher, the editors and the reviewers. Any product that may be evaluated in this article, or claim that may be made by its manufacturer, is not guaranteed or endorsed by the publisher.
